# Denosumab vs. Zoledronic Acid for Metastatic Bone Disease: A Comprehensive Systematic Review and Meta-Analysis of Randomized Controlled Trials

**DOI:** 10.3390/cancers17030388

**Published:** 2025-01-24

**Authors:** Benjamin G. Wajda, Leah E. Ferrie, Annalise G. Abbott, Golpira Elmi Assadzadeh, Michael J. Monument, Joseph K. Kendal

**Affiliations:** 1 Department of Surgery, Section of Orthopaedic Surgery, University of Calgary, Calgary, AB T2N 1N4, Canada; 2 Cumming School of Medicine, University of Calgary, Calgary, AB T2N 1N4, Canada; 3 Arnie Charbonneau Cancer Institute, University of Calgary, Calgary, AB T2N 1N4, Canada; 4 McCaig Bone & Joint Institute, University of Calgary, Calgary, AB T2N 1N4, Canada

**Keywords:** zoledronic acid, denosumab, metastatic bone disease, bone metastasis, multiple myeloma, “bone cancer”

## Abstract

**Background**: Metastatic bone disease (MBD) presents significant challenges in patient management, leading to skeletal-related events (SREs), compromised health-related quality of life, and heightened pain experiences. Denosumab (Dmab) and zoledronic acid (ZA) are bone-modifying agents (BMAs) commonly employed to mitigate the sequelae of MBD. Previous meta-analyses have assessed primary outcomes such as overall survival, pathological fractures, radiation to bone, and the time to SREs within studies. However, a single comprehensive analysis comparing their efficacy across multiple primary and secondary outcomes, as well as cost-effectiveness in specific cancer types, has not yet been conducted. **Methods**: A literature search identified relevant randomized controlled trials (RCTs), and the primary outcomes included overall survival, pathologic fractures, radiation to bone, and the time to SREs within studies. Secondary outcomes included adverse events, pain, analgesia usage, quality of life, and cost. **Results**: Meta-analysis revealed that Dmab effectively reduced the need for bone-targeted radiation therapy and was superior to ZA in delaying the time to SREs, except in multiple myeloma. Dmab also reduced pathological fracture incidences in breast cancer patients by 39%. **Conclusions**: Our analysis suggests that while both agents similarly impact overall survival and disease progression, Dmab offers advantages in SRE reduction and improved HRQoL and pain outcomes with lower rates of opioid usage, albeit with higher risks of hypocalcemia and osteonecrosis in some subgroups. The consensus on cost-effectiveness is mixed and varies based on the cancer type and healthcare system, with some studies favoring Dmab’s superior efficacy and safety, while others find ZA more cost-effective due to its lower cost. This study underscores the potential of Dmab as a preferred BMA for MBD management, especially for high-risk skeletal complications, while highlighting cancer-specific safety considerations. Further research is warranted to refine cancer-specific BMA use and optimize MBD management strategies.

## Simple Summary

This meta-analysis and systematic review compared denosumab (Dmab) and zoledronic acid (ZA) for managing bone metastases from advanced solid tumors and multiple myeloma, analyzing seven RCTs with 7441 patients. Both treatments had comparable outcomes for overall survival and disease progression. Dmab showed advantages in reducing skeletal-related events (SREs), delaying time to SREs, and reducing pathological fractures, particularly in breast cancer patients. It was also associated with fewer renal toxicities and acute phase reactions but increased risks of hypocalcemia and osteonecrosis of the jaw. Health-related quality of life improvements were observed with Dmab, alongside reduced analgesia use in some cancer types. Cost-effectiveness analyses revealed higher costs for Dmab, with marginal gains in quality-adjusted life years (QALYs). Notably, the cost-effectiveness of both agents is heavily influenced by healthcare system structures, drug pricing, and cancer type. While Dmab may benefit high-risk patients, ZA remains a cost-effective alternative in many settings.

## 1. Introduction

Bone metastases are common in advanced cancers, with incidence rates as high as 75% in breast and prostate cancer and 36% in lung cancer [1]. Five-year survival rates are reported as 50% in multiple myeloma, 20% in breast cancer, and 25% in prostate cancer; however, patients are living longer as treatments continue to improve [1,2,3]. Metastatic bone disease (MBD) presents significant challenges in patient management and often leads to devastating skeletal-related events (SREs), such as spinal cord compression, the use of radiation therapy, and pathologic fractures, often necessitating orthopedic surgical intervention. With the metastasis of primary cancers to the bone, osteolytic or osteoblastic (or mixed) lesions are formed. Late-stage bone metastasis has been characterized as a ’vicious cycle’, wherein a positive feedback loop disrupts bone homeostasis as tumor cells proliferate and dominate. Through several secreted factors, this loop causes tumor cells to increasingly stimulate osteoclastic activity [4]. SREs are associated with significant morbidity, impaired mobility, heightened patient pain experiences, increased opioid analgesic use, and compromised quality of life [5]. MBD remains a substantial burden on healthcare systems, accounting for approximately one-fifth of oncologic treatment costs in the United States—an estimated USD 40 billion annually [6].

Bone-modifying agents (BMAs), specifically bisphosphonates and more recently denosumab (Dmab), are therapeutic bone-targeting agents that have been utilized to treat skeletal complications associated with MBD to improve quality of life and survival and reduce pain and pathologic fractures. Bisphosphonates traditionally have been the most utilized agents to reduce the number of SREs in patients suffering from MBD, and zoledronic acid (ZA) has been the standard of choice as it has shown superiority among all bisphosphonates in reducing the number of SREs in patients with multiple myeloma or bone metastases from advanced cancers (breast, prostate, or solid tumors) and improving survival outcomes [7,8]. With a high affinity for hydroxyapatite crystals, ZA is taken up in bone minerals, and when internalized by osteoclasts it functions to inhibit bone resorption [9,10]. As ZA works to shift metabolic activity in favor of bone formation, causing increased bone mass [10], the overall application of ZA has been partially limited due to associated adverse events such as renal impairment, osteonecrosis of the jaw (ONJ), and acute-phase reactions [11,12]. In 2010, a novel monoclonal antibody (Dmab) emerged. Dmab is an inhibitor of the receptor activator of nuclear factor- κB ligand (RANKL) and works to prevent bone breakdown. Clinical trials [13,14,15,16] have shown Dmab to be superior to ZA in reducing the time to first SRE in MBD patients while being reported as more effective than ZA in mitigating other adverse outcomes such as renal toxicity and acute phase reactions [17].

To date, the comparative efficacy of Dmab versus ZA has been primarily reported in the context of SREs and other adverse events [18]. Furthering the previous work by Chen et al. [18] which evaluated the efficacy and tolerability of Dmab versus ZA in the delay of SREs in MBD patients, we report a comprehensive comparison of Dmab versus ZA from randomized controlled trials that also evaluates efficacy profiles while additionally assessing pain outcomes, analgesic use, and health-related quality of life (HRQoL) by primary cancer types in MBD. A cost-effectiveness analysis of Dmab versus ZA in the management of MBD is also performed, with the aim of informing providers to guide clinical decision-making.

## 2. Materials and Methods

### 2.1. Literature Search

The systematic review and meta-analysis was conducted in accordance with the PRISMA 2020 guidelines (PROSPERO ID: CRD42024509041) [19]. A literature search identified relevant studies using the MEDLINE and EMBASE databases up to 15 February 2024. Search terms used included the following: “zoledronic acid”, “denosumab”, “metastatic bone disease”, “bone metastases”, “multiple myeloma”, “cancer”, or a combination of these terms. The population included patients with bone metastases secondary to advanced solid tumors or multiple myeloma. The studies included were restricted to articles that were available in English and available online. A manual search was also performed by two individual reviewers (B.W. and L.F.).

### 2.2. Inclusion Criteria

The identified studies were included in the meta-analysis if the following criteria were met: (1) target population: patients with bone metastasis secondary to advanced solid tumors or multiple myeloma; (2) intervention: Dmab versus ZA; (3) a report of at least one of the following outcomes: (a) the incidence of an SRE, (b) overall survival, (d) overall disease progression, (e) pain scores, (f) quality of life, and (g) adverse events; (4) methodological criteria: randomized controlled double-blind trials (RCTs); and (5) involved human patient populations. Studies utilizing overlapping data sets were included, but only unique data sets were included in the meta-analysis. Studies analyzing the cost-effectiveness of Dmab compared to ZA in bone metastases were included if these were analyses utilizing bone complication rates that were estimated from published phase III studies and modified to reflect real-world incidence. Case reports, editorials, letters to the editors, reviews, non-English articles, and studies with no comparison group were excluded for both the systematic review and meta-analysis. Two independent reviewers (B.W. and L.F.) individually assessed studies according to the pre-determined inclusion criteria using *Covidence* (Veritas Health Innovation, Melbourne, Australia). A third reviewer was present to settle any disagreements that arose (A.A.).

### 2.3. Data Extraction

The primary outcomes of interest included overall survival, overall disease progression, pathologic fracture, radiation to bone, the time to first SRE within the study, and time to first and subsequent SREs within the study. Secondary outcomes included pain, pain interference, analgesic use, HRQoL, and cost. Adverse events were assessed using the Common Terminology for Criteria for Adverse Events (CTCAE) version 3.0 [20]. Infectious AEs were based on the Medical Dictionary for Regulatory Activities System Organ Class categorization infections and infestations (MedDRA v12.0 and v12.1). The Brief Pain Inventory-Short Form (BPI-SF) was used to assess pain severity and pain interference during routine functional activities. BPI-SF scores ranged from 0 to 10 and the following defined intervals: 1–4 indicated mild pain, 5 or 6 indicated moderate pain, and 7 to 10 indicated severe pain [21]. Pain interference ranged from 0 (no interference) to 10 (total interference) [22]. The degree of analgesic use was assessed using the Analgesic Quantification Algorithm (AQA) score. Data were extracted individually by the authors into a Microsoft Excel (Microsoft Corp., Redmond, WA, USA) spreadsheet, and any discrepancies between reviewers were resolved with discussion.

Time-to-event data included the following: the time to first SRE within the study, the time to first and subsequent SRE within the study, the time to worsening pain in patients with no/mild pain at baseline, and the time to increased pain interference (aggregate) in patients with no pain at baseline. Dichotomous data included the following: all AEs, serious AEs, fatal AEs, Common Terminology Criteria for Adverse Events (CTCAE) grade > 3 AE, an AE leading to medication (Rx) discontinuation, renal toxicity/renal AEs, infectious AEs, osteonecrosis of the jaw (ONJ), an AE associated with an acute phase reaction, and hypocalcemia.

### 2.4. Quality Assessment

The quality of the studies was assessed using the Cochrane Risk of Bias tool. Specifically, the studies were evaluated for the following elements: sequence generation, allocation concealment, blinding of participants and personnel, blinding of outcome assessors, incomplete outcome data, selective outcome reporting, and other sources of bias.

### 2.5. Statistical Analysis

Data synthesis and statistical analysis were performed using the R (Version 4.4) software. Time-to-event data were pooled as a hazard ratio (HR) and dichotomous data sets were pooled as a risk ratio (RR). Meta-analysis was performed using random and fixed-effects models to pool effect sizes across studies. The inconsistency index (I^2^) and Chi-squared test were used to quantify heterogeneity. Significant heterogeneity was defined as *p* < 0.10 or I^2^ > 50%. Statistical significance was defined as *p* value < 0.05. The findings of the meta-analysis are shown as forest plots. In time-to-event forest plots, the Ln [HR] values are reported within the figure and converted to the HR for ease of comparison within the text. The pooled analysis was performed according to PRISMA guidelines [19].

## 3. Results

### 3.1. Study Selection

In accordance with the inclusion and exclusion criteria, 2614 studies were initially identified from the MEDLINE and Embase databases for the meta-analysis. *Covidence* (Veritas Health Innovation, Melbourne, Australia) was used to assist in removing duplicates and screening studies. A total of 313 duplicate studies were removed, resulting in 2301 studies that were screened. Screening of abstracts and titles resulted in 65 studies that were further assessed for eligibility. Of the 65 studies, 58 were excluded for being either the incorrect intervention type, study design, publication type, or inability to access the full text. Ultimately, seven studies were identified and included in our meta-analysis.

**Figure 1:**
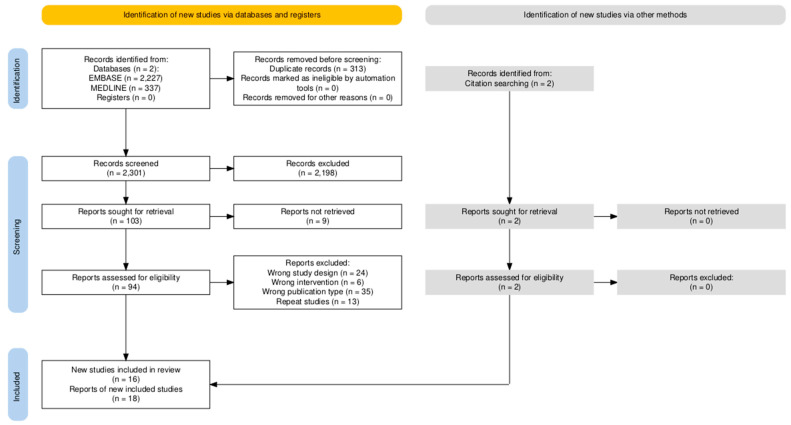
The PRISMA flow diagram illustrating the study selection process for meta-analysis and systematic review of Dmab versus ZA.

Using our initial search strategy, a second search was designed to retrieve articles analyzing the cost effectiveness of Dmab compared to ZA. For the systematic review on cost analysis, of the 2301 studies originally identified, 41 studies on cost-effectiveness were further assessed for eligibility and nine studies met inclusion criteria. Two additional studies which met the inclusion criteria were identified through citation searching. The PRISMA flow diagram depicting the study selection process is shown in Figure 1 [23].

### 3.2. Study Characteristics

We identified seven studies that met our inclusion criteria, as shown in Table 1. Of those studies, only four RCTs used unique/original data sets to compare the efficacy of ZA versus Dmab for use in patients with MBD, while the other three utilized patient data from the four unique RCTs [13,14,15,16]. Of the four RCTs, the sample size ranged from 1718 to 2046 patients. Each one of the four studies included patients with different malignancies: (1) breast, (2) prostate, (3) multiple myeloma, and (4) all other cancers, including multiple myeloma and excluding prostate and breast (“All, excluding breast and prostate”). A total of 7441 individual patients were enrolled in these studies, with 3721 patients assigned to the Dmab group and 3720 patients assigned to the ZA group. In each trial, 120 mg of Dmab was administered subcutaneously (SC) every 4 weeks and 4 mg of ZA was administered intravenously (IV) every 4 weeks. A summary of the study characteristics of the included studies is shown in Table 1.

### 3.3. Quality Assessment of Included Studies

**Figure 2:**
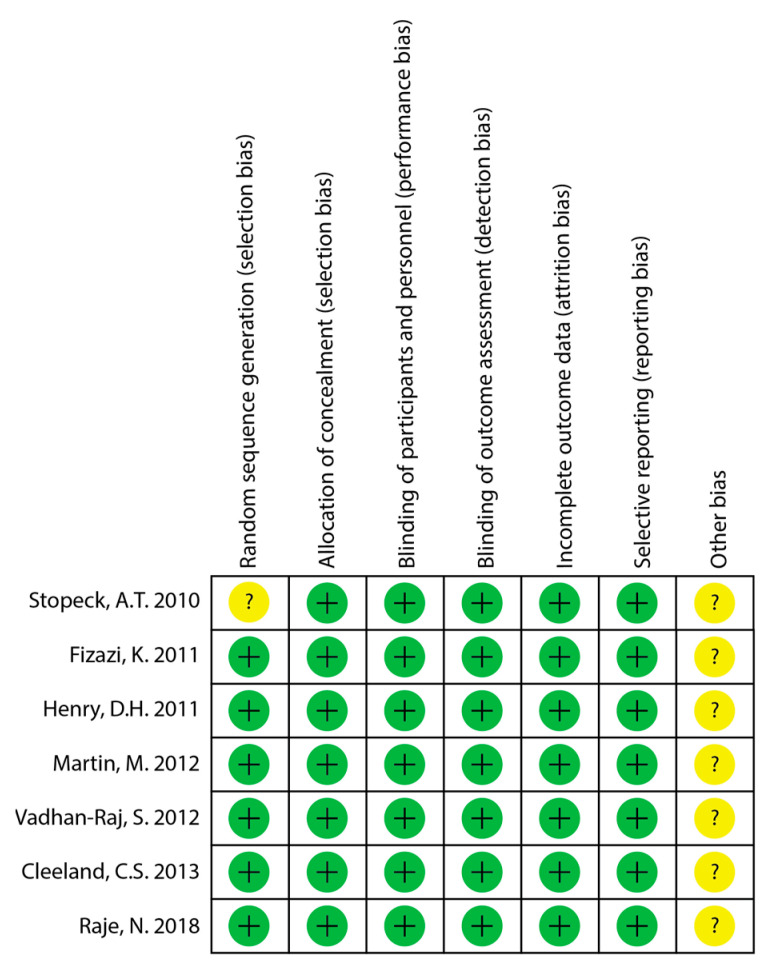
Quality assessment of included studies for risk of bias [13,14,15,16,24,25,26]. “+” = low risk of bias; “?” = minimal information and cannot judge risk of bias.

The quality of the included studies was assessed using the Cochrane Risk of Bias 1 tool. The quality of the all of the included RCTs was reported as high, though Stopeck et al. does not explicitly describe the method used to generate the random sequence [13]. The quality assessment for risk of bias is summarized in Figure 2.

### 3.4. Skeletal Related Events (SREs)

**Figure 3:**
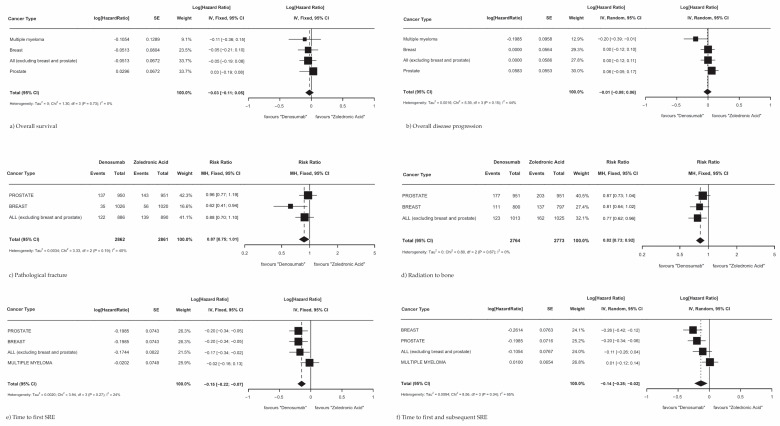
Skeletal-related events in MBD patients treated with Dmab vs. ZA. (**a**) Overall survival, (**b**) overall disease progression, (**c**) pathological fracture, (**d**) radiation to the bone, (**e**) time to first on-SRE within study, (**f**) time to first and subsequent SRE within study.

Overall survival, overall disease progression, the time to first SRE within the study, and the time to first and subsequent SRE within the study were available from four studies with a combined 7441 patients [13,14,15,16]. The incidence of pathologic fracture and radiation to the bone was only reported in three studies with 5087 patients [13,14,15]. Only two studies defined overall disease progression; Fizazi et al. [14] described overall disease progression as encompassing visceral distant metastatic disease, locoregional progression, biochemical progression, and excluding adverse events, and Raje et al. [16] assessed disease progression on the basis of the International Myeloma Working Group uniform response criteria [27]. In a pooled analysis of all primary cancer types, there was no significant difference observed in overall survival (Figure 3a; HR 0.97; 95% CI 0.90–1.05; *p* = 0.46) or overall disease progression (Figure 3b; HR 0.99; 95% CI 0.92–1.06; *p* = 0.83) between patients treated with Dmab and patients treated with ZA. When analyzed by primary cancer subtype, Dmab was favored in reducing disease progression in multiple myeloma patients (HR 0.82; 95% CI 0.68–0.99).

Treating patients with Dmab resulted in significantly fewer patients requiring radiation therapy to the bone (Figure 3d; RR 0.82; 95% CI 0.73–0.92; *p* < 0.01) compared to patients treated with ZA. There was no significant reduction in pathologic fractures across all cancer types (Figure 3c; RR 0.87; 95% CI 0.75–1.01; *p* = 0.06), though Dmab did show a significant reduction in pathological fractures in breast cancer patients with a reduction in likelihood of 39% (RR 0.61; 95% CI 0.39–0.94). Additionally, Dmab was significantly superior to ZA across all cancer subtypes studied in delaying the time to first SRE within the study (Figure 3e; RR 0.86; 95% CI 0.80–0.93; *p* < 0.01) and in delaying the time to first and subsequent SRE within the study (Figure 3f; RR 0.87; 95% CI 0.78–0.98; *p* = 0.03). When analyzing SREs by each primary cancer subtype, Dmab was found to be more effective in delaying the time to first and subsequent SRE than ZA for all primary cancers (RR 0.87; 95% CI 0.78–0.98), though was not more effective than ZA in multiple myeloma (RR 1.01; 95% CI 0.89–1.15). Most pooled effects did not show significant heterogeneity, with overall survival and radiation to the bone showing no to low heterogeneity and all other outcomes showing moderate heterogeneity, except for time to first and subsequent SRE which showed considerable heterogeneity.

### 3.5. Adverse Events

The risk ratios (RRs) of the AEs comparing Dmab versus ZA groups from the included RCTs [13,14,15,16] are presented in Table 2. Individual forest plots comparing AEs are available in the Supplementary Materials. Dmab was superior to ZA with a decreased risk of any adverse event (RR 0.98; 95% CI 0.97–1.00; *p* = 0.018), renal toxicity/renal-related events (RR 0.63; 95% CI 0.54–0.75, *p* < 0.0001), and AEs associated with acute phase reaction (RR 0.47; 95% CI 0.36–0.62; *p* < 0.0001). Dmab had a higher risk of ONJ (RR 1.43; 95% CI 1.03–1.97; *p* = 0.032) and hypocalcemia (RR 1.97; 95% CI 1.62–2.39; *p* < 0.0001) compared to ZA.

**Figure 4:**
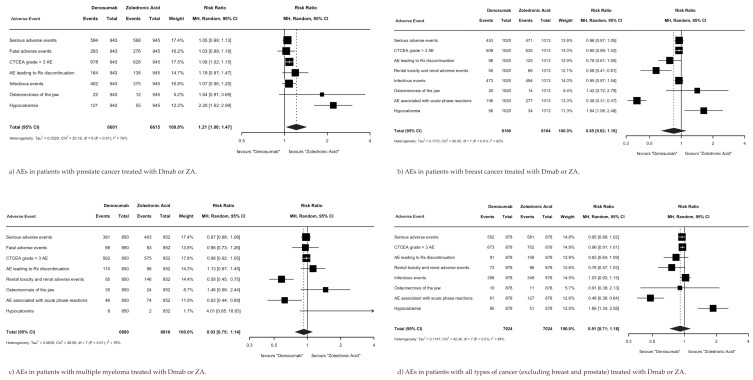
Summary of AEs by primary cancer type.

The risk ratios of AEs in patients with MBD treated with Dmab versus ZA were assessed within four primary cancer types, prostate (Figure 4a), breast (Figure 4b), multiple myeloma (Figure 4c), and all cancers, excluding breast and prostate (Figure 4d), using data from four original RCTs [13,14,15,16]. There was no significant difference in total AEs between MBD patients with primary breast cancer (RR 0.85; 95% CI 0.62–1.16; *p* = 0.30), multiple myeloma (RR 0.93; 95% CI 0.75–1.14; *p* = 0.48), or all cancers excluding breast and prostate (RR 0.91; 95% CI 0.71–1.18; *p* = 0.49). MBD patients with primary prostate cancer had significantly fewer AEs when treated with ZA versus Dmab (RR 1.21; 95% CI 1.00–1.47; *p* = 0.048).

Within the primary prostate cancer subgroup (Figure 4a), patients treated with ZA versus Dmab had significantly fewer events of hypocalcemia (RR 2.20; 95% CI 1.62–2.99; *p* < 0.001) and Dmab showed no superiority in reducing any AEs. Within the primary breast cancer subgroup (Figure 4b), patients treated with Dmab versus ZA had significantly fewer renal toxicity/renal AEs (RR 0.58; 95% CI 0.61–1.00; *p* = 0.001) and AEs associated with acute phase reactions (RR 0.38; 95% CI 0.31–0.47; *p* < 0.001), but Dmab led to more events of hypocalcemia (RR 1.64; 95% CI 1.08–2.48; *p* = 0.02). Within the primary multiple myeloma cancer subgroup (Figure 4c), patients treated with Dmab versus ZA had significantly fewer renal toxicity/renal AEs (RR 0.58; 95% CI 0.45–0.75; *p* < 0.001) and AEs associated with acute phase reactions (RR 0.62; 95% CI 0.44–0.89; *p* = 0.01), and ZA did not exhibit superiority in the reduction in any AEs. Finally, within all cancers group, excluding the breast and prostate group (Figure 4d), patients treated with Dmab versus ZA had significantly fewer AEs associated with acute phase reactions (RR 0.48; 95% CI 0.36–0.64; *p* < 0.001), but ZA was superior to Dmab in reducing AEs related to hypocalcemia (RR 1.86; 95% CI 1.34–2.58; *p* < 0.001).

### 3.6. Pain-Related Outcomes

**Figure 5:**
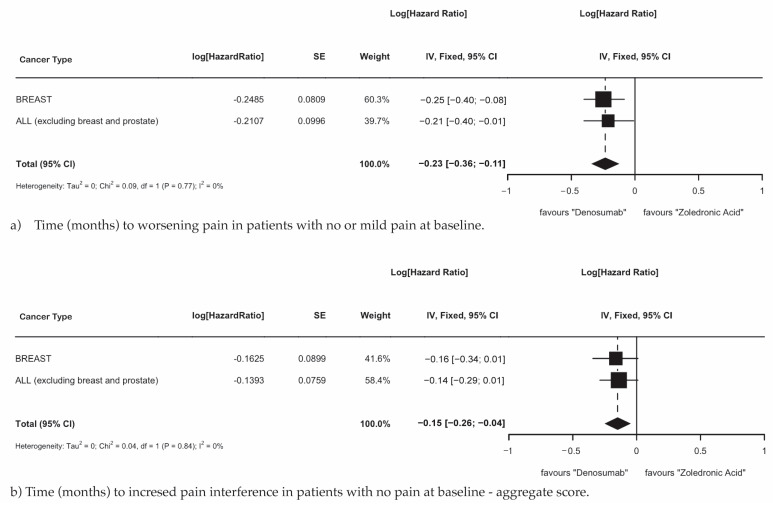
(**a**) Time (months) to worsening pain in patients with no or mild pain at baseline. (**b**) Time (months) to increased pain interference in patients with no pain at baseline—aggregate score.

MBD patients with primary breast cancer and MBD patients with all cancers, excluding breast and prostate cancer, demonstrated that treatment with Dmab was superior to ZA in delaying the time to worsening pain in patients with no or mild pain at baseline (Figure 5a; HR 0.70; 95% CI 0.70–0.90; *p* = 0.0002) and the aggregate time to increased pain interference in patients with no pain at baseline (Figure 5b; HR 0.86; 95% CI 0.77–0.96; *p* = 0.010).

### 3.7. Cost Assessment

Forty-one initial articles and abstracts in the search were identified. Of the 41 articles, nine were identified to meet the inclusion criteria, two additional studies were identified by citation searching (Table 3). All studies used the Markov decision model with study horizons ranging from 1 year to a lifetime, and probabilities for calculations and transitions among health states were derived from previously completed phase III clinical trials. Each study utilized the same treatment comparison of 120 mg of Dmab SC and 4 mg of ZA IV, administered monthly. Six of the studies were conducted in the USA using a US payer perspective, four studies [28,29,30,31,32,33] were conducted in Europe [34,35,36,37], and one study was conducted in India [38]. All studies utilized a payer perspective. The cancer subtypes with bone metastasis included breast cancer [29,30,31,34,35,36,38], castrate-resistant prostate cancer [28,30,34,35,36,38], multiple myeloma [33,37], and other solid tumors (OSTs) [30,34,35,36]. All cost values were converted to USD and the US Consumer Price Index (CPI) was used to adjust for inflation to September 2024 dollar values. Fewer patients experiencing SREs was demonstrated for Dmab in comparison to ZA in all studies and all cancer subtypes, except for in the OST subgroup (0.06), with SRE differences ranging from −0.99 to 0.06 [30]. In all studies except for Lothgren et al., Dmab had higher mean costs (inclusive of drug administration costs and SRE costs) than ZA. The cost per quality-adjusted life years (QALYs) gained was substantially variable, ranging from USD 21,365.03 [34] to USD 5,643,804.04 [39].

## 4. Discussion

### 4.1. Efficacy and Safety Profile of Denosumab Versus Zoledronic Acid

This meta-analysis of seven RCTs based on four unique data sets evaluated a total of 7441 patients and suggests that while Dmab and ZA offer similar effects on overall survival and disease progression across multiple cancer types, there are some differences in specific outcomes related to SREs. Dmab demonstrated a greater capacity to reduce the need for radiation therapy to the bone by 18% but did not lower the incidence of pathological fracture across all cancer types in the pooled analysis. However, among breast cancer patients, the incidence of pathologic fracture was reduced by 39%, with statistical significance. These results are in concordance with previous meta-analyses comparing Dmab to ZA [18,40]. Multiple myeloma is also quite sensitive to both systemic therapy and radiation, which may negate the effects of BMAs as the primary treatment modalities (chemotherapy and radiotherapy) are highly effective at reducing local disease burden and preventing skeletal destruction. ZA is particularly effective in managing osteolytic lesions due to its multifaceted mechanism of action, which involves anti-angiogenic effects and the inhibition of osteoclast-mediated bone resorption through the inhibition of essential GTPase signaling proteins that are essential for osteoclast function, while Dmab does not exhibit the same anti-angiogenic effects on osteoclasts [41].

The lack of significant differences in overall survival and disease progression between Dmab and ZA suggests that while both agents are effective in reducing bone complications, neither drug demonstrates superiority over the other when it comes to overall survival. This finding underscores the palliative role of bone-modifying agents in managing bone metastases rather than altering cancer prognosis. Confounders such as disease severity, underlying disease heterogeneity, and the influence of concurrent anti-neoplastic agents could contribute to the lack of consistent impact on survival and progression outcomes. Despite most outcomes displaying low to moderate heterogeneity, the considerable heterogeneity observed in time to first SRE within the study and time to first and subsequent SREs may indicate differences in response based on individual cancer subtypes or other patient-specific factors, warranting further investigation into these potential variations. Dmab’s favorable profile in reducing fracture risk and delaying SREs, combined with its administration advantages, may support its use as a preferred treatment option, particularly for patients at high risk of bone complications.

Patients treated with Dmab compared to those treated with ZA had fewer overall AEs, renal toxicity/renal-related events, and AEs associated with acute phase reactions, but a higher incidence specifically of ONJ and hypocalcemia, which is consistent with the previously published literature [17,18]. It should be noted that there was significant heterogeneity observed between studies for the following outcomes: overall AEs, CTCAE grade >3, an AE leading to treatment discontinuation, and an AE associated with an acute phase reaction. As reported, over 95% of patients experienced an AE on Dmab or ZA, for which we believe this high rate can be attributed to the inclusive nature of the CTCAE severity grading scale of 1–5. As further described in the CTCAE v5.0, Grade 1 includes AEs that are asymptomatic or mild symptoms, clinical or diagnostic observations only, and instaces where the intervention is not indicated [42]. Therefore, any symptoms that fall within a Grade 1 AE, for example “mild fatigue”, are included, elevating the total number of AEs reported. In addition, fatal adverse events were reported to be approximately 20% in both the Dmab and ZA groups, which was surprisingly high. Within individual RCTs, Raje et al. reported fatal AEs as treatment-emergent (rather than drug-related), and they attributed deaths to patient’s baseline comorbidities [16]. Fizazi et al. did not comment on whether fatal AEs were treatment-emergent or drug-emergent [14]. When looking at efficacy based on the cancer subgroup, Dmab demonstrated superiority in reducing AEs compared to ZA in MBD patients with primary breast cancer, multiple myeloma, and all other cancers (excluding breast and prostate cancer); however, ZA was superior to Dmab in reducing AEs in patients with primary prostate cancer. The use of Dmab also demonstrated superiority to ZA in reducing the time to first SRE across all primary cancer subgroups. This observation is in accordance with previously published studies [18,39,43,44]. Interestingly, although ZA resulted in fewer AEs in the prostate group, Dmab was effective in reducing the time to first SRE in patients with primary prostate cancer.

Pain-related outcomes were more challenging to evaluate, as they were not consistently reported across RCTs, resulting in a sample size that was too small to reliably evaluate in the overall MBD population. As a result, conclusions could only be drawn for specific primary cancer types. MBD patients with primary breast cancer and all other cancers (excluding breast and prostate cancer) demonstrated that Dmab was superior in delaying the time to worsening pain and the time to increased pain interference compared to patients treated with ZA. This finding is consistent with results in the previously reported literature [18].

Only two of seven RCTs assessed analgesic use in MBD patients receiving Dmab versus ZA [25,26]. As a result, we were unable to complete a meta-analysis due to the lack of comparative data. However, as previously reported by Cleeland et al., fewer patients treated with Dmab shifted from no/low analgesic use (AQA scores 0–2) to strong opioid use (AQA scores 3–7) compared with patients treated with ZA (relative difference, 20%, absolute difference, 2%) [26]. Similarly, Vadhan-Raj et al. reported that in ZA-treated patients, there was a 27% relative increase in patients who shifted to strong opioid analgesic use compared to Dmab-treated patients [25]. For patients with no or low analgesic use at baseline, the median time to initiation of strong opioid analgesics was not reached in the denosumab group, compared to 29.5 months in the zoledronic acid group (HR 0.90; 95% CI, 0.75–1.08; *p* = 0.27) [26]. These findings are supportive of Dmab’s superiority to ZA in minimizing pain.

With respect to HRQoL, this outcome was similarly only assessed in two of seven RCTs [24,25] as there was insufficient data to complete a meta-analysis. Martin et al. reported that an average of 10% more patients in the Dmab-treated group compared to the ZA-treated group had a meaningful improvement in HRQoL (defined as a greater or equal to a 5-point increase in the FACT-G total score) [24]. On average, the Dmab treatment group had 7% fewer patients with worsening HRQoL within the study compared to the ZA-treatment group. Martin et al. also observed that Dmab improved HRQoL regardless of the pain severity at baseline compared to ZA, with a 14% improvement in patients with no/mild pain at baseline and a 9% improvement in patients with moderate/severe pain at baseline [24]. In contrast, Vadhan-Raj et al. reported no significant difference in HRQoL scores for patients treated with Dmab versus ZA [25]. These findings are suggestive of Dmab’s superiority in improving the quality of life in patients suffering from MBD.

### 4.2. Cost Assessment

Eleven reviewed studies demonstrate that both denosumab (Dmab) and zoledronic acid (ZA) effectively reduce skeletal-related events (SREs), though cost-effectiveness findings vary. Dmab generally incurs higher total costs, with differences influenced by the cancer type, country, and time horizon. Six studies [28,29,31,32,36,38] found Dmab to be more effective in reducing SREs but not cost-effective compared to ZA, even at high willingness-to-pay thresholds, as the higher costs of Dmab do not outweigh the similar benefits observed with ZA. Conversely, five studies [30,34,35] supported Dmab as being cost-effective due to its superior efficacy, safety, and administration ease. European studies [34,35] and one U.S. study [30] reported a lower cost per QALY for Dmab, ranging from USD 21,365.03 to USD 33,952.65, while Xie et al. [31] reported the highest cost per QALY of USD 5,643,804.04 for prostate cancer. The large range in cost per QALY observed (USD 21,365.03 to USD 5,643,804.04) arises primarily from two “outlier” studies (Xie et al. [31] and Snedecor et al. [28]) evaluating prostate cancer. Along with methodological differences, the finding that ZA was superior to Dmab in reducing adverse events in prostate cancer likely contributed to these high-cost estimates. Two recent studies [33,37] assessed the cost-effectiveness of Dmab for multiple myeloma. Across four European countries, the average cost per QALY was USD 28,800.12, meeting WHO cost-effectiveness thresholds. In the U.S., Raje et al. [33] reported a cost per QALY of USD 136,376.39 from a societal perspective. Both studies used a lifetime horizon, supporting their conclusions, though reliance on solid tumor data and extrapolated survival estimates beyond clinical trial follow-up periods introduced some uncertainty.

Wide cost-per-QALY ranges stem from methodological differences, including time horizons, variable cost inputs, SRE utility assumptions, and drug discontinuation rates. Shorter time horizons inflate costs; for example, extending a model’s horizon from 27 to 60 months reduced the cost per QALY from USD 950,369 to USD 264,834. SRE utility assumptions vary widely: Xie et al. [31,32] limited SRE effects to 13 weeks and assumed only one SRE occurred per cycle, while others modeled effects over a year or incrementally with numerous SREs, which is more reflective of real-world conditions [25,26]. Utility inputs were derived using methods like time trade-off interviews [30] or EQ-5D data, though EQ-5D’s limitations, such as ceiling effects, may affect accuracy. Drug discontinuation was modeled using Phase III trial data [29,30,34,36] or exponential decay [31], while other studies did not mention discontinuation rates [34,40]. Cost inputs also varied; for instance, the cost of surgery to the bone ranged from USD 20,734 [29] to USD 29,792 [30] in breast cancer. European studies reported lower costs per QALY due to reduced incremental drug costs, while U.S. studies showed higher costs, driven by Dmab’s administration expenses. Overall, differences in methodologies, assumptions, and regional cost structures significantly influence cost-effectiveness outcomes.

## 5. Conclusions

This meta-analysis highlights the distinct therapeutic profiles of Dmab and ZA for managing SREs in patients with MBD. While both agents show comparable effects on overall survival and disease progression, Dmab demonstrates greater efficacy in reducing specific SREs, such as radiation therapy to the bone and pathological fractures in breast cancer, and prolongs SRE-free intervals in most cancers.

Dmab’s favorable safety profile, including fewer renal events and acute-phase reactions, along with its convenience and ability to delay pain progression, offers clinical advantages, despite higher costs. Further research is needed to deepen our understanding of pain management and HRQoL, and to address methodological variances in cost-effectiveness analyses to refine our understanding of these agents’ economic value and optimize decision-making for MBD management, considering both clinical and economic factors.

## Figures and Tables

**Table 1:** Characteristics of studies meeting our inclusion criteria comparing Dmab 120 mg SC versus ZA 4 mg IV every 4 weeks. N/A = not applicable.

	Authors	Year	Primary Cancer	Original Data Set	Study Duration (Months)	Sample Size	Median Age	% Female	Outcomes Measured
1.	Stopeck, A.T. [13]	2010	Breast	N/A	34	ZA: 1020 Dmab: 1026	ZA: 56 Dmab: 57	ZA: 99.1 Dmab: 99.2	SREs AEs
2.	Fizazi, K. [14]	2011	Prostate	N/A	41	ZA: 951 Dmab: 950	ZA: 71 Dmab: 71	ZA: 0 Dmab: 0	SREs AEs
3.	Henry, D.H. [15]	2011	All, excluding breast and prostate	N/A	34	ZA: 890 Dmab: 886	ZA: 61 Dmab: 60	ZA: 38 Dmab: 34	SREs AEs
4.	Martin, M [24]	2012	Breast	Stopeck, A.T. 2010 [13]	34	ZA: 1020 Dmab: 1026	ZA: 56 Dmab: 57	ZA: 99.1 Dmab: 99.2	HRQoL
5.	Vadhan-Raj, S. [25]	2012	All, excluding breast and prostate	Henry, D.H. 2011 [15]	34	ZA: 890 Dmab: 886	ZA: 61 Dmab: 60	ZA: 38 Dmab: 34	Pain severity Pain interference Analgesic use (AQA) HRQoL
6.	Cleeland, C.S. [26]	2013	Breast	Stopeck, A.T. 2010 [13]	34	ZA: 1020 Dmab: 1026	ZA: 56 Dmab: 57	ZA: 99.1 Dmab: 99.2	Pain severity Pain interference Analgesic use (AQA)
7.	Raje, N. [16]	2018	Multiple myeloma	N/A	42	ZA: 859 Dmab: 859	ZA: 63 Dmab: 63	ZA: 46 Dmab: 45	SREs AEs

**Table 2:** Summary of adverse events from included RCTs.

Adverse Events	Dmab n/N	ZA n/N	RR (95% CI)	*p*-Value
Any adverse event *	3550/3691 (96.2%)	3597/3688 (97.5%)	0.98 (0.97, 1.00)	0.018 *
CTCAE grade > 3 AE	2522/3691 (68.3%)	2540/3688 (68.9%)	0.99 (0.94, 1.05)	0.79
AE leading to Rx discontinuation	463/3691 (12.5%)	470/3688 (12.7%)	0.97 (0.79, 1.20)	0.80
Serious adverse events	1990/3691 (53.9%)	2023/3688 (54.9%)	0.98 (0.93, 1.04)	0.53
Fatal adverse events	372/1793 (20.7%)	369/1797 (20.5%)	1.01 (0.89, 1.14)	0.87
Renal toxicity/renal adverse events *	208/2748 (7.6%)	328/2743 (12.0%)	0.63 (0.54, 0.75)	<0.0001 *
Infectious events	1233/2841 (43.4%)	1218/2836 (42.9%)	1.01 (0.94, 1.09)	0.78
ONJ *	87/3691 (2.4%)	61/3688 (1.7%)	1.43 (1.03, 1.97)	0.032 *
Hypocalcemia *	280/3691 (7.6%)	142/3688 (3.9%)	1.97 (1.62, 2.39)	<0.0001 *
AE associated with acute phase reaction *	213/2748 (7.8%)	478/2743 (17.4%)	0.47 (0.36, 0.62)	<0.0001 *

* Denotes significance (*p*-value ≤ 0.05). n = adverse events, N = total sample size.

**Table 3:** Description for cost-effectiveness analyses of Dmab and ZA in patients with bone metastases.

Author (Year)	Disclosures	Country	Study Horizon	Primary Cancer	Mean Costs (Drug Administration and SRE $USD)			SRE Difference	QALY Difference	Cost per SRE Avoided ($USD)		Cost per QALY Gained ($USD)
					Dmab	ZA	Difference			Dmab	ZA	
Cristino et al. (2017) [34]	Amgen	Czech Republic	Lifetime	Prostate Breast OST	29,583.92 50,087.89 26,242.88	27,725.34 48,016.06 24,834.79	1858.58 2071.83 1408.09	−0.63 −0.73 −0.27	0.09 0.09 0.04	3015.26 2890.11 5272.45	REF REF REF	21,365.03 22,804.18 33,952.65
Lothgren et al. (2013) [35]	Amgen	Austria, Sweden, Switzerland	12 months	Prostate Breast OST	14,038.35 12,916.67 17,313.63	16,870.82 15,437.45 19,917.11	−2832.47 −2520.78 −2603.48	−0.17 −0.15 −0.14	NR	NR	NR	NR
Snedecor et al. (2012) [28]	Novartis	USA	27 months	CRPC	46,143.51	34,825.59	11,317.92	−0.24	0.007	12,736.81	16,114.43	1,528,216.55
Snedecor et al. (2013) [29]	Novartis	USA	27 months	Breast	43,393.78	33,135.34	10,258.44	−0.30	0.0102	7269.10	10,179.06	950,369.40
Stopeck et al. (2012) [30]	Novartis	USA	Lifetime	CRPC Breast OST	106,290.35 150,832.08 68,188.36	96,689.12 132,139.62 62,525.45	9601.23 18,692.46 5662.91	−0.81 −0.99 −0.06	0.14 0.17 0.06	11,905.31 18,839.77 14,609.61	REF REF REF	68,656.68 109,665.86 94,401.72
Wadhwa et al. (2024) [38]	N/A	India	Lifetime	Breast	12,350.99	6219.23	6131.76	−0.28	0.013	25,137.06	REF	485,558.49
Xie et al. (2011) [31]	Novartis	USA	12 months 36 months	Prostate Prostate	51,012.19 100,272.07	39,734.69 80,271.93	11,277.50 20,000.14	−0.20 −0.28	NR	102,522.37 74,075.29	REF	5,643,804.04 3,998,295.95
Xie et al. (2012) [32]	Novartis	USA	12 months	Breast	41,777.23	32,704.84	9072.39	−0.06	NR	159,452.60	REF	NR
Yfantopoulos et al. (2013) [36]	Novartis	Greece	14.5 months 22.5 months 9 months	Prostate Breast OST	11,990.11 18,909.51 9380.67	10,797.27 17,815.43 8811.26	1192.84 1094.08 569.41	−0.10 −0.12 −0.10	0.013 0.012 0.005	28,309.21 31,757.76 35,267.12	REF REF REF	354,926.75 499,241.68 587,333.47
Raje et al. (2018) [33]	Amgen	USA	Lifetime	Multiple Myeloma	410,284.74	347,526.18	62,758.56	−0.05	0.244	NR	NR	136,376.39
Terpos et al. (2019) [37]	Amgen	Austria, Belgium, Greece, Italy	Lifetime	Multiple Myeloma	174,864.93	137,084.02	37,780.91	−0.05	0.215	NR	NR	28,800.12

NR = Not reported. REF = Reference. All values expressed in USD and price adjusted for inflation to September 2024 values.

## Data Availability

The data that support the findings of this study are available from the corresponding author upon reasonable request.

## Supplementary Materials

The following supporting information can be downloaded at https://www.mdpi.com/article/10.3390/cancers17030388/s1, Figure S1: Adverse events from included RCTs, differentiated by cancer type.
