# Supplementary material for: Denosumab vs. Zoledronic Acid for Metastatic Bone Disease: A Comprehensive Systematic Review and Meta-Analysis of Randomized Controlled Trials
Source: Cancers (Basel). 2025 Jan 24;17(3):388. doi: 10.3390/cancers17030388 (PMC11816125; doi:10.3390/cancers17030388)
Supplement: Supplementary file 1 [file cancers-17-00388-s001.zip › cancers-3414386-supplementary.pdf]

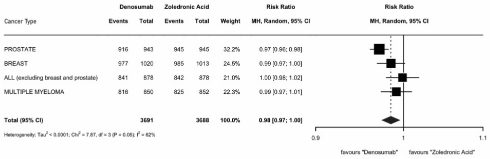

a) Any adverse event

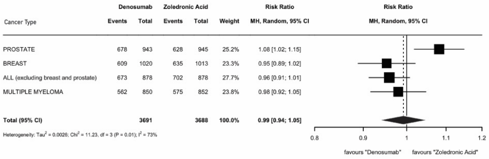

b) CTCAE grade > 3 AE

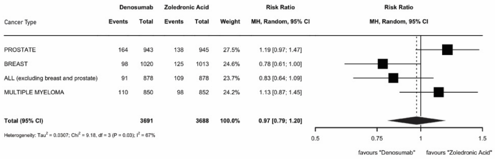

c) AE - leading to treatment (Rx) discontinuation

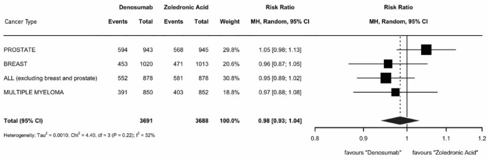

d) Serious adverse events

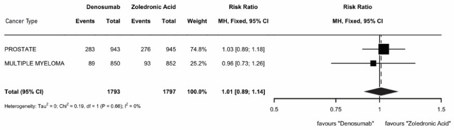

e) Fatal adverse events

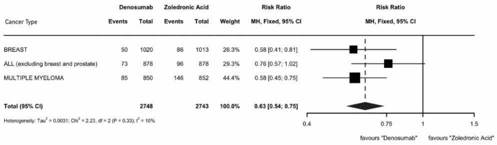

f) Renal toxicity / renal adverse events

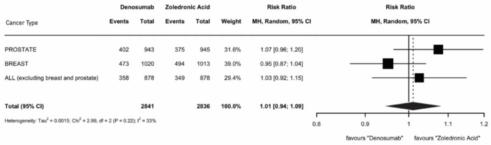

g) Infectious events

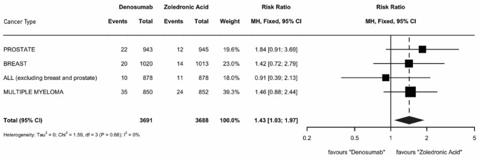

h) ONJ

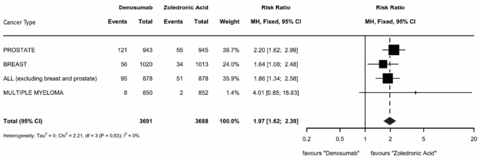

i) Hypocalcaemia

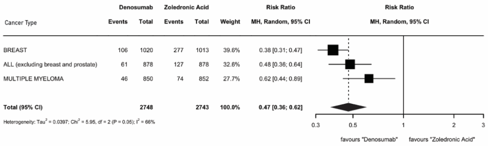

j) AE-associated with acute phase reaction

Figure S1. Adverse events from included RCTs, differentiated by cancer type.
